# CD4+ cell count recovery in naïve patients initiating cART, who achieved and maintained plasma HIV-RNA suppression

**DOI:** 10.7448/IAS.17.4.19481

**Published:** 2014-11-02

**Authors:** Dominique Costagliola, Jean-Marc Lacombe, Jade Ghosn, Constance Delaugerre, Gilles Pialoux, Lise Cuzin, Odile Launay, Amélie Ménard, Pierre de Truchis, Murielle Mary-Krause, Laurence Weiss, Jean-François Delfraissy

**Affiliations:** 1 INSERM and Sorbonne Universités, UPMC Paris Univ 06, Pierre Louis Institute, UMR-S 1136, Paris, France; 2 AP-HP, Hotel-Dieu, Université Paris Descartes, Paris, France; 3 AP-HP, Hôpital Saint-Louis, Université Paris Diderot et INSERM, Paris, France; 4 AP-HP, Hôpital Tenon, Service de Maladies Infectieuses, Paris, France; 5 CHU Toulouse, Service de Maladies Infectieuses, Toulouse, France; 6 AP-HP, Hôpital Cochin, Fédération d'Infectiologie, Paris, France; 7 AP-HM, Hôpital de la Conception, Service de Maladies Infectieuses, Marseille, France; 8 AP-HP, Hôpital Raymond Poincaré, Service de Maladies Infectieuses, Garches, France; 9 AP-HP, Hôpital Européen Georges Pompidou, Institut Pasteur, Paris, France; 10 Agence Nationale de Recherches sur le Sida et les Hépatites Virales, Paris, France

## Abstract

**Introduction:**

A key objective of combined antiretroviral therapy (cART) is to reach and maintain high CD4 cell counts to provide long-term protection against AIDS-defining opportunistic infections and malignancies, as well as other comorbidities. However, a high proportion of patients present late for care. Our objective was to assess CD4 cell count recovery up to seven years in naïve patients initiating cART with at least three drugs in usual clinical care.

**Methods:**

From the French Hospital Database on HIV, we selected naïve individuals initiating cART from 2000 with at least two years of follow-up. Participants were further required to have achieved viral load suppression by six months after initiating cART and were censored in case of virological failure. We calculated the proportion of patients (Kaplan-Meier estimates) who achieved CD4 recovery to >500/mm^3^ according to baseline CD4 cell count.

**Results:**

A total of 15,025 patients were analyzed with a median follow-up on ART of 65.5 months (IQR: 42.3–96.0). At cART initiation, the median age was 38.6 years (IQR: 32.2–46.0), 9734 (64.8%) were men, median CD4 cell count was 239 (IQR: 130–336) and 2668 (17.8%) had a prior AIDS event. Results are presented in the Table 1.

**Conclusions:**

This study shows that CD4 cell counts continue to increase seven years after cART initiation, whatever the baseline CD4 cell count. Failing to achieve CD4 recovery with continuous viral load suppression is rare for naïve patients initiating cART in routine clinical practice, but takes substantially longer in patients who initiate antiretroviral therapy at low CD4 cell counts.

**Table 1:** KM estimates of achieving a CD4 cell count >500/mm^3^ over time according to baseline CD4 cell count at cART initiation

Baseline CD4+cell count, Median (IQR)	Year 1	Year 2	Year 3	Year 4	Year 5	Year 6	Year 7
CD4<200 (*n*=5909), 100 [39–156]	8% (7–9)	21% (20–22)	33% (31–34)	43% (42–45)	52% (50–54)	60% (58–61)	61% (59–62)
200≤CD4<350 (*n*=5751), 269 [235–305]	40% (39–42)	61% (60–62)	73% (72–74)	81% (580–82)	85% (84–86)	88% (87–89)	90% (89–91)
350≤CD4<500 (*n*=2252), 404 [374–443]	74% (72–76)	87% (85–88)	91% (90–92)	94% (93–95)	95% (93–96)	96% (95–98)	97% (95–98)

